# Pediatric Beta Blocker Therapy: A Comprehensive Review of Development and Genetic Variation to Guide Precision-Based Therapy in Children, Adolescents, and Young Adults

**DOI:** 10.3390/genes15030379

**Published:** 2024-03-20

**Authors:** Mollie Walton, Jonathan B. Wagner

**Affiliations:** 1 Ward Family Heart Center, Kansas City, MO 64108, USA; 2 Division of Clinical Pharmacology, Toxicology and Therapeutic Innovation, Children’s Mercy, 2401 Gillham Road, Kansas City, MO 64108, USA; 3 Department of Pediatrics, University of Missouri-Kansas City School of Medicine, Kansas City, MO 64108, USA

**Keywords:** beta-blockers, pediatrics, pharmacogenomics

## Abstract

Beta adrenergic receptor antagonists, known as beta blockers, are one of the most prescribed medications in both pediatric and adult cardiology. Unfortunately, most of these agents utilized in the pediatric clinical setting are prescribed off-label. Despite regulatory efforts aimed at increasing pediatric drug labeling, a majority of pediatric cardiovascular drug agents continue to lack pediatric-specific data to inform precision dosing for children, adolescents, and young adults. Adding to this complexity is the contribution of development (ontogeny) and genetic variation towards the variability in drug disposition and response. In the absence of current prospective trials, the purpose of this comprehensive review is to illustrate the current knowledge gaps regarding the key drivers of variability in beta blocker drug disposition and response and the opportunities for investigations that will lead to changes in pediatric drug labeling.

## 1. Introduction

Beta adrenergic receptor antagonists (i.e., beta blockers) are classified as first generation (e.g., propranolol), which are non-selective for the antagonism of the β1 and β2 receptor; second generation (e.g., metoprolol), with relative selectivity for the β1 receptor; and third generation (e.g., carvedilol), which block β1-, β2-, and α1-receptors. Beta blockers are frequently prescribed medications for adult-onset cardiac diseases, such as hypertension, atrial arrhythmias, and chronic heart failure. Likewise, this anti-arrhythmic medication has been recognized as a first-line agent for many pediatric tachyarrhythmias, both in the non-operative and peri-operative settings [1]. Despite frequent utilization in the pediatric population, there remains a dearth of pediatric-specific data to inform dose individualization and precision-based care to this population [2,3]. Most pediatric therapeutic decisions have been extrapolated from adult experiences. In pediatrics, commonly used enterally administered beta blockers include atenolol, carvedilol, metoprolol, and propranolol. Therefore, the purpose of this review is to extensively examine the existing literature related to beta blocker utilization in children, the relevant pathways that may lead to variability in response, and opportunities for future investigations.

## 2. Dose–Exposure–Response Paradigm and Pediatric Trials

Implementing precision therapeutics in the care of the individual pediatric cardiovascular patient relies on a distinct understanding of the dose–exposure–response relationship of the administered drug in order to predict response for a particular dose administered. That understanding is dependent upon knowledge of the unique drug properties (e.g., protein binding affinity coefficient, physicochemical properties, drug preparation) and patient factors (e.g., age, underlying cardiac pathophysiology, variable drug metabolism or transport) that contribute to the drug’s “systemic exposure” for the individual child. Consequently, this “systemic exposure” (e.g., dose–exposure) can be directly related to drug response, collectively known as the dose–exposure–response relationship. For example, clopidogrel, a P2Y purinoreceptor 12 (P2RY_12_) receptor antagonist that prevents platelet aggregation, requires cytochrome P450 2C19 (CYP2C19)-mediated bioactivation to produce its active metabolite [4,5]. In those with gene variation associated with a loss of function (e.g., CYP2C19*2), a reduction in the systemic exposure of the active moiety is observed [6]. Consequentially, this leads to inadequate exposure at the drug target (e.g., P2RY_12_ receptor) to adequately prevent adenosine diphosphate-induced platelet aggregation (i.e., response) [7,8]. This was validated in a systematic review by Mega et al. [8]. In this scenario, the altered response may not be secondary to a drug–target abnormality (e.g., *P2RY_12_* gene variants) [9], but rather a drug metabolism (dose–exposure) problem leading to the aberration in response.

**Figure 1:**
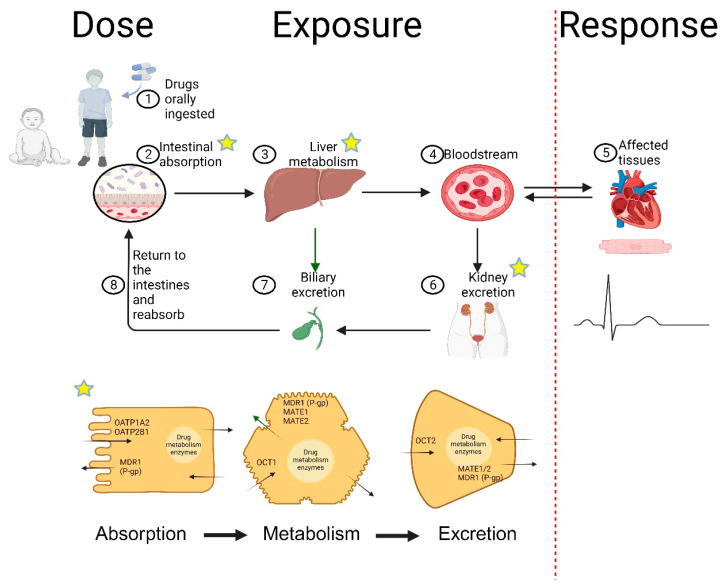
**Dose–Exposure–Response Pathway.** Red dashed line delineates the dose–exposure (drug disposition) relationship. Figure created with Biorender.com. Abbreviations: MATE—Multidrug and Toxin Extrusion, MDR1—Multidrug Resistance Protein 1, MRP2—multidrug resistance-associated protein 2, OATP—organic anion transporter polypeptides, OCT—organic cation transporter, P-gp—P-glycoprotein.

In pediatric pharmacotherapy, it is known that extrapolation of the adult experience to a “small adult size” is complicated by age-related differences in the pharmacokinetics of drugs in children [3,10]. For example, CYP2C19 enzymatic development (i.e., ontogeny), predominantly occurring during infancy, where 50 to 75% of adult levels are not achieved until at least 5 months of age [11], would be expected to impact the CYP2C19-mediated metabolism of clopidogrel amongst neonates and young infants. Thus, ontogenic factors influencing the drug disposition pathway (absorption, distribution, metabolism, and elimination: ADME) must likewise be taken into consideration when depicting the dose–exposure relationship in a child. Collectively, the assiduousness in advancing precision-guided therapy for pharmacotherapeutics must incorporate a thorough evaluation of the drug’s disposition pathway from drug absorption through elimination (e.g., dose–exposure relationship), as demonstrated in Figure 1.

In addition to a thorough examination of the patient factors and drug properties influencing the dose–exposure relationship, attention must be paid to factors driving variable drug response beyond abnormalities in the dose–exposure relationship. These factors of abnormal response could be categorized by two distinct mechanisms, namely, (1) altered drug-to-drug receptor engagement, possibly secondary to genetic variation leading to protein aberrations in the drug receptor, or (2) altered downstream drug target signaling cascade following a sufficient drug–target interaction. Systematic evaluations concerning the influence of ontogeny and genetic variation on the response pathways (i.e., drug targets and associated signaling cascade pathways) are likewise needed in conjunction with the dose–exposure factors to adequately inform precision-guided pharmacotherapy.

## 3. Evolution of Beta Blocker Therapy in Children

Beta blockers are a class of medication with U.S. Food and Drug Administration approval for very few indications in pediatrics; yet, they are commonly used for various off-label indications [12]. Following the discovery in the mid-twentieth century of beta blockers as primary regulators of heart rate and myocardial contractility in response to catecholamine stimulation [13], propranolol, a small molecular substrate antagonizing this receptor to counter these effects [14], was discovered and became the first commercially available beta adrenergic receptor antagonist (i.e., “beta blocker”) in the United States. The use of beta blockers for the treatment of adults with heart failure was supported by studies conducted by Bristow et al. [15] that demonstrated the downregulation and desensitization of beta adrenergic receptors in response to chronic catecholamine stimulation in failing human heart tissue and experimental heart failure models [16]. Studies between the 1970s and 2000s demonstrated clinical efficacy and improvement in morbidity and mortality with the use of beta blocker therapy in the management of adult patients with heart failure [17]. As such, beta blockers remain a mainstay in the medical therapy of adults with heart failure.

Owing to pediatric heart failure being relatively uncommon, with an annual incidence of about 1.1 per 100,000 children per year [18], and associated difficulty in performing clinical trials, treatment recommendations in pediatric patients with heart failure were thus extrapolated from the results of clinical trials in adults. Unfortunately, the same results in improvement in survival in pediatric patients have not been demonstrated [19,20]. This differential response was further emphasized by the carvedilol trial published in 2007, which showed that there was no improvement in clinical outcomes among children with heart failure treated with placebo versus carvedilol [21]. It is important to note that various factors may have led to this observed difference, including the differences in the etiologies of pediatric heart failure (commonly dilated cardiomyopathy and congenital heart disease) compared with adults (primarily ischemic heart disease) and the heterogeneous nature of the children enrolled, influencing the efficacy of the drug, along with the relatively small sample size. A Cochrane review of beta blockers for heart failure in children published in 2020 [22] reported that there was not enough evidence to support or discourage the use of beta blockers in children with congestive heart failure or to propose a pediatric dosing scheme.

Beta blockers are also commonly used for the management of pediatric tachyarrhythmias, such as supraventricular tachycardia (SVT), some forms of ventricular arrhythmias, and long QT syndrome. SVT is the most common tachyarrhythmia, requiring treatment in pediatrics [23]. Currently, comparative randomized clinical trials assessing the management of SVT are scarce, and there is limited consensus and evidence to guide antiarrhythmic agent selection in the management of pediatric arrhythmias, including SVT [24]. In 2006, a survey study involving pediatric cardiologists and pediatric electrophysiologists showed that there is no consensus regarding the appropriate agent to use in the management of SVT in infants, although propranolol was the most commonly used [1]. As there is little evidence to drive agent selection in the management of SVT, there is also limited predictability in knowing what antiarrhythmic agent, dose, or combination of agents may achieve rhythm control. Overall, the available evidence demonstrates only 50% of pediatric SVT quiescence with monotherapy, suggesting that determining the most effective, minimal dose exposure (i.e., dose optimization) is needed for this population [25,26,27].

Infantile hemangiomas are common soft-tissue tumors that can develop during childhood. Historically, systemic glucocorticoids were the mainstay of treatment for complicated hemangiomas [28] until 2008, when a paradigm shift occurred wherein propranolol was repurposed in the regression of infantile hemangiomas [29]. With this anecdotal experience, propranolol became first-line therapy, despite the lack of randomized, controlled clinical trials and a lack of pediatric formulation [30]. In 2015, a phase 2–3 clinical trial demonstrated the efficacy of a pediatric-specific oral propranolol in the treatment of infantile hemangiomas requiring systemic therapy, utilizing a dose of 3 mg/kg/day for 6 months in the treatment [31]. The ability of propranolol to cross the blood–brain barrier (with potential unproven long-term neurocognitive effects), as well as the pharmacokinetic variability of propranolol [32], prompted the investigation of nadolol, a synthetic nonselective beta blocker with no known intrinsic sympathomimetic activity. In this trial, oral nadolol was non-inferior to propranolol in the treatment of infantile hemangioma [33]. However, these data have not been extensively translated into clinical practice.

Marfan’s syndrome is a connective tissue disorder with cardiac implications, including progressive aortic root dilation and potential aortic dissection, the leading cause of death in Marfan’s syndrome [34]. After a small, randomized trial published in 1994 comparing propranolol and no pharmacologic therapy showed a reduced rate of aortic root dilation among treated adult patients, beta blockers became the mainstay of the medical management of patients with Marfan’s syndrome [35]. With the discovery of transforming growth factor β dysregulation in the pathogenesis of some aortic aneurysms, the angiotensin receptor blockade was subsequently proposed as an alternative treatment to attenuate aortic root growth progression in Marfan syndrome. In 2014, a prospective, randomized trial conducted by the Pediatric Heart Network over a three-year period showed no significant difference in the rate of aortic-root dilation between children and young adults with Marfan syndrome treated with losartan (an angiotensin II type 1-receptor blocker) and atenolol [34]. However, there was substantial variability in the response between treatment groups. Post-hoc analyses by van Driest et al. identified protein aberrations secondary to genetic variation that may have led to this variability and are described further below [36].

## 4. Contributions of Ontogeny and Genetic Variation in Beta Blocker Disposition and Response

The dearth of pediatric-specific data related to pediatric cardiovascular drug disposition and response reduces the ability to develop precision-based dosing algorithms in developing cardiovascular patients. Given the common utilization of beta blocker therapy in pediatric cardiovascular patients, prospective guidelines need to be generated to maximize efficacy and minimize the potential for an adverse event (i.e., dose optimization). These recommendations to deliver dose optimization for the developing child must be informed with strong data that characterize the dose–exposure–response relationship in the pediatric cardiovascular patient. Prospective investigations characterizing the entire range of the dose–exposure–response relationship must consider the existing literature related to the impact of the ontogeny and genetic variation of the relevant drug disposition and response pathways. Therefore, the intention of the remainder of this review is to present three essential points that must be considered when acquiring knowledge related to variability in beta blocker disposition and response in the developing cardiac patient. This approach has been previously utilized to detect knowledge deficits linked to the contribution of ontogeny and genetic variation on drug disposition and response in children [37,38].

## 5. Fundamental Issues for Evaluating Variability in Drug Disposition in Pediatrics

*Knowledge of gene products is quantitatively important in the disposition (absorption, distribution, metabolism, and excretion) and the response of beta blocker therapy.*

The majority of the enterally administered beta blockers utilized in pediatrics are atenolol, carvedilol, metoprolol, and propranolol. Despite these aforementioned agents sharing a commonality of antagonism of human β1 adrenergic receptors (ADRB1), these agents vary in physicochemical profile (e.g., octanol–water partition coefficient, protein binding affinity). It is incredibly important to note that these differences influence the overall disposition (i.e., ADME) of the drug substrate and reflect the differences that must be considered when discovering relevant proteins that may influence the drug’s systemic exposure. For example, carvedilol, metoprolol, and propranolol are lipophilic, with propranolol having the highest degree of lipophilicity [39]. However, atenolol is a hydrophilic drug substrate and, theoretically, could be more dependent on transporter-mediated distribution or transcellular movement [40]. Alternatively, propranolol, being the most lipophilic of these agents, would be expected to easily translocate across cellular membranes (i.e., passive diffusion).

Equally as important to drug disposition is the degree of protein binding. Owing to its hydrophilic nature, atenolol is not extensively protein-bound [41]. However, carvedilol and propranolol are extensively protein bound, ~98% and 95%, respectively [42,43,44]. Metoprolol is only 10% protein-bound in systemic circulation [45]. Propranolol preferentially binds to α1-acid glycoprotein [46], whereas carvedilol and metoprolol preferentially bind to albumin [42]. Collectively, these extensively protein-bound beta blockers (e.g., carvedilol, propranolol) may be more affected by decreased binding, either due to the diminished expression of albumin or α1-acid glycoprotein expression or the displacement of the drug secondary to physiologic conditions, such as hyperbilirubinemia or free fatty acids, which are known to be higher in newborns relative to older children [10,47].

Interestingly, protein binding affinity can be stereoselective, with the R(+) propranolol enantiomer preferentially binding to albumin and the S(−) propranolol enantiomer preferentially binding to α1-acid glycoprotein [48]. The racemic mixture of beta blockers further provides a complexity that must be accounted for when characterizing the dose–exposure–response relationship, as there are stereoselective routes for drug disposition described below. Additionally, the R(+) and S(−) enantiomers can have differential effects on adrenergic receptors. For example, R(+) carvedilol has no appreciable beta adrenergic antagonism but can antagonize α-1 adrenergic receptors, resulting in more vasodilation [49]. Conversely, S(−) carvedilol has more β1 adrenergic antagonism and a similar magnitude of α-1 adrenergic antagonism [49]. Only S(−) propranolol has any appreciable β-1 antagonism [50], and, therefore, any disruptions to S(−) propranolol could result in either an enhanced response (e.g., poor metabolism of S(−) propranolol or preferential metabolism of only the R(+) enantiomer) or a lack of response (e.g., rapid metabolism of S(−) propranolol). Collectively, these drug properties must be considered when evaluating the drug disposition and response pathways for each beta blocker. Herein, we describe the drug disposition process in further detail, with notable pathways summarized in Table 1.

## 6. Absorption

Owing to its hydrophilic nature, atenolol is dependent on transporter-mediated absorption from the gastrointestinal lumen [40], with organic anion transporter polypeptides (OATPs) 1A2, 2B1, and organic cation transporter (OCT) 1 being noted as potential proteins involved with enterocyte uptake [51,52,53]. The utilization of the targeted inhibitors of transporters provides great opportunities to acquire knowledge gaps and validate transporter-mediated transcellular uptake. At the level of the enterocyte, the greater the reduction in area under the curve (AUC) with a concurrent inhibitor administration, the larger the contribution of that transporter’s role in transcellular drug uptake, as reduced uptake into the enterocyte is accompanied by a decrease in circulating plasma drug concentrations. Alternatively, if there is not a significant disruption in the total AUC in the presence of a known inhibitor, less dependency on that specific transporter is required for enterocyte uptake. As such, concurrent administration of known inhibitors of OATP1A2 and OATP2B1 (e.g., orange juice, apple juice) has demonstrated a 40% to 80% reduction in atenolol’s total AUC, a common variable to determine systemic exposure [52,54]. However, in vitro data from Mimura et al. demonstrated very little atenolol OATP1A2 and OATP2B1 cellular uptake relative to OCT1, where a four-fold increased uptake relative to OATP1A2 and OATP21 was noted [53]. Furthermore, in the presence of flavonoids, atenolol uptake was two to three-fold lower in stably expressed OCT1 cells [53]. Collectively, these in vitro data suggest that OCT1 may have a larger role in intestinal absorption for atenolol; however, these data need to be replicated more broadly in vivo. The absolute bioavailability of atenolol is ~50% [55,56,57], and this is partially secondary to its affinity to Multidrug Resistance Protein 1 (MDR1), otherwise known as P-glycoprotein (P-gp), which is responsible for efflux transport back into the intestinal lumen from the enterocyte [58]. Alternatively, carvedilol, metoprolol, and propranolol are moderately to highly lipophilic and, subsequently, are extensively absorbed from the gastrointestinal tract via passive diffusion [42,59,60]. Consequently, there is not any significant transporter-mediated transcellular distribution at the enterocyte or hepatocyte level for these aforementioned substrates. However, carvedilol, metoprolol, and propranolol are only ~25–30%, ~50%, and 25% bioavailable, respectively, due to the substantial first pass effect [42,59,60].

## 7. Distribution

Entry into the hepatocyte after absorption of carvedilol, metoprolol, and propranolol predominantly occurs via passive diffusion as noted above. However, atenolol, given its hydrophilic nature, is subject to transporter-mediated hepatic uptake, as noted above in *Absorption*. In vitro screening of candidate transporters demonstrated that OCT1 was associated with the highest magnitude of atenolol transcellular uptake and OCT2 to a lesser extent [53]. Given its tissue expression predominantly on the basolateral surface of the liver, OCT1 potentially could have a larger role in atenolol hepatic uptake relative to enterocyte uptake [61]. In contrast to the paradigm noted above for enterocyte uptake, inhibitors that significantly affect hepatic uptake would result in increased systemic exposure (i.e., AUC) and, thereby, the greater increase in AUC with concurrent inhibitor administration would validate the greater importance of that particular transporter in hepatic transcellular uptake. Therefore, the functional importance of OCT1-mediated atenolol uptake was verified by inhibitory studies, including the known OCT1 substrates (e.g., flavonoids) noted above, where atenolol uptake was significantly attenuated and could have applicability regarding hepatic OCT1-mediated uptake [53]. Additionally, quinidine, a known OCT1 inhibitor [62], attenuated atenolol transcellular uptake [53], further confirming the importance of OCT1 in atenolol distribution. The influence of the concurrent administration of OCT1 substrates in human subjects and, more specifically, human hepatic uptake, remains unknown but requires further elucidation. Likewise, the influence of OCT2, predominantly expressed on the basolateral surface of renal tubule cells [61], on atenolol distribution and subsequent excretion remains unknown. Although in vitro data have suggested that atenolol undergoes transcellular uptake via OCT2, there was two to three-fold less transport efficiency relative to OCT1 [63]. Finally, there is no evidence to suggest the preferential stereoselectivity of the R(+) or S(−) enantiomers related to OCT1/2 binding affinity and subsequent transcellular uptake [64].

## 8. Metabolism

To date, it has been demonstrated that phase 1 metabolism (e.g., CYP-mediated) of the aforementioned beta blockers occurs predominantly via a CYP2D6-mediated process [65,66,67,68,69], with CYP1A2 contributing to the metabolism of carvedilol and propranolol in addition to CYP2D6 [65,67,69]. The exception includes atenolol, which does not undergo extensive phase 1 or 2 metabolism, and, in fact, ~90–100% of the atenolol is excreted and unchanged in the urine [56,70,71]. Although CYPs 2B6, 2C9, and 3A4 are capable of metabolizing these drug substrates, CYP1A2 and CYP26 are responsible for the majority of drug biotransformation. For metoprolol, the majority of metabolism (~70%) occurs via CYP2D6 [72], with o-demethylation composing the main route of metabolism [73,74]. The stereoselectivity of these routes can occur with preferential o-demethylation for the R(+) enantiomer [74] and α-hydroxylation for the S(−) enantiomer [75]. The importance of CYP2D6-dependent metabolism was demonstrated by a ~60% reduction in metabolism with a concurrent administration of a potent CYP2D6 inhibitor (e.g., quinidine) in vivo [72]. For carvedilol, aromatic ring oxidation occurs via CYP2D6 [65,69,76] and side chain oxidation via CYP1A2 [65,69]. Demethylation contributes minimally to carvedilol metabolism; however, this process is catalyzed predominantly by CYP2C9 [77]. A stereoselective metabolism is observed with carvedilol as well as R(+) carvedilol metabolized by CYP2D6 and a minor contribution from CYPs 3A4 and 1A2 [65]. S(−) carvedilol is predominantly metabolized by CYP1A2 with a minor contribution from CYP26 and CYP3A4 [65]. In vitro data demonstrate that the concurrent administration of known CYP2D6 antidepressants, including sertraline, fluvoxamine, and bupropion, resulted in a ~64%, 25%, and 15% decreased aromatic ring oxidation to 4-hydroxylphenylcarvedilol [78]. This is confirmed in vivo, where the concurrent administration of fluoxetine and carvedilol resulted in a 77% increase in R(+) carvedilol AUC and no significant increase in S(−) carvedilol AUC [79], validating CYP2D6 contribution to the CYP-mediated metabolism of R(+) carvedilol. For propranolol, two oxidation reactions contribute to the majority of propranolol metabolism. Side-chain oxidation, contributing to ~40–45% of propranolol biotransformation, occurs preferentially via CYP1A2, with a minor contribution from CYP2D6 [67,80]. This oxidative process is a two-step process involving the aforementioned CYP enzymes catalyzing propranolol to N-desisopropylpropranolol as the rate-limiting step, which is further catalyzed to naphthoxylactic acid via monoamine oxidase (MAOA) and mitochondrial aldehyde dehydrogenase (ALDH2) [80,81]. Ring oxidation, contributing to ~40–45% of propranolol biotransformation, occurs preferentially via CYP2D6 with a minor contribution from CYP1A2, resulting in the development of a 4-hydroxypropranolol metabolite [67,80]. Differences in propranolol clearance have been noted based on sex, with males having a 63% higher clearance predominantly due to enhanced clearance via side chain oxidation due to a known higher expression of CYP1A2 in males than in females [81,82]. Similar to the aforementioned beta blockers, stereoselective drug metabolism for the R(+) and S(−) enantiomers has been reported [83]. In fact, stereoselective ring oxidation occurs with R(+) enantiomer and side chain oxidation favoring the S(−) enantiomer [84].

The phase 2 metabolism of the carvedilol and propranolol occurs predominantly via conjugation with uridine 5′-diphospho (UDP) glucuronosyl transferases (UGTs) [85,86,87,88]. UGTs 1A1, 2B4, and 2B7 are the primary enzymes involved in glucuronidation of carvedilol [87] with a preference for the S(−) enantiomer in liver microsomes [86]. UGTs 1A9, 1A10 (enterocyte) 2B4, and 2B7 are the primary enzymes responsible for propranolol glucuronidation [85] and are similar to carvedilol in the stereoselectivity of S(−) enantiomer glucuronidation [88]. Albeit a minimal contribution, sulfation can also occur with the 4-hydroxypropranolol metabolite via SULT1A3 with a preference for the R(+) enantiomer [89]. Overall, the contribution of the UGT- and SULT-mediated metabolism of these drug substrates is minimal in comparison to the aforementioned CYPs.

## 9. Excretion

Data regarding beta blockers and their affinity for efflux transporters are limited. However, in vitro, atenolol has been identified as a substrate for the Multidrug and Toxin Extrusion (MATE) 1 [63], an efflux transporter on the apical membranes of hepatocytes and kidney proximal tubule cells [90]. Atenolol is a substrate for MATE2 [63], an efflux transporter isolated to the apical membranes of kidney proximal tubule cells [90]. Likewise, atenolol is a known substrate for the MDR1 (P-gp), a protein diffusely expressed in intestinal, liver, and kidney tissues [90] in vitro that potentially contributes to its excretion into the intestinal lumen, bile canaliculus, or proximal kidney tubule [63]. Similarly, the available evidence demonstrates that carvedilol is a substrate for MDR1 in addition to the multidrug resistance-associated protein 2 (MRP2) [91]. In a small cohort of human participants, those treated with rifampin, known to upregulate MDR1 and MRP2 expression, had a significantly higher expression of MDR1 and MRP2 mRNA and, subsequently, was correlated with lower carvedilol AUC [91].

Collectively, OATP/OCT uptake and MATE/MDR1 efflux transporters appear to be critical elements of atenolol disposition. Conversely, CYP1A2 and CYP2D6, and, to a lesser extent, CYP2C9 and CYP3A4, contribute to the disposition of carvedilol, metoprolol, and propranolol.

## 10. Response

**Figure 2:**
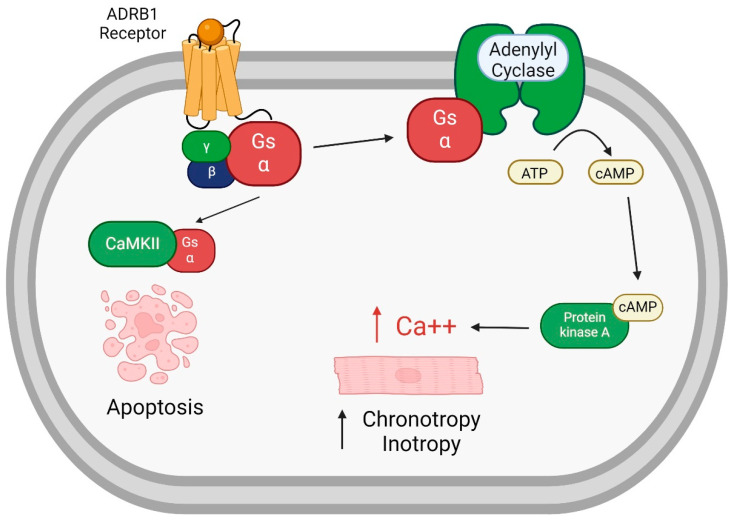
**Beta Adrenergic Signaling Cascade.** Activation of ADRB1 results in a signaling cascade leading to enhanced chronotropy and inotropy. Additionally, it can stimulate calmodulin-dependent protein kinase II (CaMKII) to result in apoptosis. Figure created with Biorender.com. Abbreviations: ADRB1—β1 adrenergic receptor, ATP—adenosine triphosphate, CaMKII—calmodulin-dependent protein kinase II, cAMP—cyclic adenosine monophosphate.

The β1 adrenergic receptor, encoded by the ADRB1 gene, is a G protein-coupled receptor as seen in Figure 2. As such, there are four molecular components involved in signal initiation by a ligand. These necessary components include the receptor itself, the heterotrimeric G protein to which it couples for the activation of downstream factors, G protein receptor kinases that regulate the receptor–G protein interactions, and regulators of G protein signaling [92]. Ligand (i.e., agonist) binding to the receptor induces a conformational change that facilitates the interaction of the receptor intracellular domains with the heterotrimeric G protein. Agonist–receptor coupling induces the dissociation of the trimeric G protein into two subunits, each of which has the capacity to modulate a signaling pathway.

Myocardial β1 adrenergic receptors are coupled through the subunit Gα_s_ to the stimulation of adenylyl cyclase and through it to activate protein kinase A, which phosphorylates contractile and calcium regulatory proteins to enhance contractility. The effects mediated through this pathway include chronotropy, inotropy, and an increase in electrical automaticity. The β1 receptor is the major adrenergic receptor expressed on cardiac myocytes (compared to α1-3, β2, and β3), and accounts for about 80% of all β-adrenergic receptors in normal adult myocardium [93].

***Knowledge of allelic variation in the genes of interest is associated with functional consequences in vivo.***

## 11. Genetic Determinants of Beta Blocker Disposition

The solute carrier organic anion transporter (*SLCO*) gene family, encoding for the OATP uptake transporters, and its effects have been well described for many drug substrates (e.g., statins, methotrexate, and valsartan) [94,95,96]. The *SLCO* family is expressed predominantly on the basolateral surface of a hepatocyte; however, it can be expressed on the basolateral surface of enterocytes, especially for *SLCO1A2* and *2B1* [90]. Collectively, these transporters are responsible for cellular uptake from the gastric lumen or systemic circulation. Gene variants associated with diminished expression and function create a scenario where the AUC can be decreased secondary to diminished absorption or increased secondary to diminished hepatic uptake/clearance. *SLCO2B1* c.1457C>T gene variation, associated with reduced drug and xenobiotic transport [97], significantly reduced fexofenadine, a known OATP2B1 substrate [98], AUC in human subjects [99], thus validating its role in diminished cellular uptake. Despite being described as an OATP2B1 substrate [51], atenolol AUC was not altered in a small cohort of human participants with *SLCO2B1* c.1457C>T gene variation [52]. This study requires replication in a larger cohort before the effect of *SLCO2B1* SNPs on atenolol systemic exposure is discounted. However, as noted above, there are conflicting data regarding the atenolol’s substrate affinity towards drug transporters, with more recent analysis suggesting the enhanced transcellular transport of atenolol with OCT1 [53]. There is a paucity of data related to the effects of *SLC22A1* (OCT1) genetic variation and its effect on atenolol systemic exposure. Available evidence with an OCT1 substrate, metformin, demonstrates that individuals with at least one *SLC22A1* gene variant (c.181C>T, rs12208357; c.1201G>A, rs34130495; Met420del, rs72552763; c.1393G>A, rs34059508) had a significantly higher AUC relative to those with the reference genotype [100]. There is currently an absence of in vitro or in vivo data evaluating the pharmacogenetic effect of *SLC22A1* gene variants on atenolol exposure; therefore, it requires further elucidation in the future.

The relationship between genes involved in phase 1 metabolism and beta blocker disposition is more robust relative to drug transporters. *CYP2D6* is a highly polymorphic gene, with over 170 allelic variants having been noted [101] within a range of metabolism activity (e.g., poor, intermediate, normal, ultrarapid) [102]. Given that carvedilol, metoprolol, and propranolol are substrates for this highly relevant and polymorphic enzyme, one could expect a wide range of systemic exposure amongst patient populations dosed with these agents, possibly resulting in an altered drug response. For example, Sehrt and colleagues demonstrated allelic-specific differences in carvedilol systemic exposure in a cohort of 110 adults with an array of active *CYP2D6* genes [103], with those with no “active” genes having three-to-four-fold higher R(+) carvedilol amounts relative to those with ≥2 “active” *CYP2D6* genes [103]. Not surprisingly, in 4-hydroxyphenyl carvedilol (4-OH-carvedilol), an active metabolite with ~13 times more potency compared to the parent drug, systemic exposure was significantly lower for those with no active genes relative to those ≥2 “active” *CYP2D6* genes. However, the *CYP2D6* genotype did not have any impact on hemodynamic markers (e.g., heart rate, blood pressure), suggesting that CYP2D6-mediated systemic variation had no influence on the response [103]. The plausible explanations for this disconnect could be that S(−) carvedilol, a more potent enantiomer, does not undergo the same magnitude of CYP2D6-mediated metabolism relative to the R(+) and, thus, is not subject to the same *CYP2D6* gene effect. Additionally, the higher AUC values for the parent drug in those with no “active” genes may be mitigated by the lower development of the highly active 4-OH-carvedilol, thus resulting in no significant difference in response amongst genotype groups. These data demonstrating a CYP2D6-mediated genetic effect on systemic exposure have been observed in other adult investigations [104,105,106] but have not been prospectively evaluated in the developing child. In children, carvedilol did not improve heart failure outcomes in a cohort of 161 children and adolescents [21]. However, there was nearly a five- and seven-fold range in steady state S(−) carvedilol trough concentrations in that pediatric cohort, implying that alterations in systemic exposure, secondary to altered drug disposition, could influence the magnitude of response in those randomized to carvedilol. *CYP2D6* genotype results in a significantly disparate systemic exposure of metoprolol for both S(−) and R(+) enantiomers [107]. This was validated in a systematic review and meta analysis by Blake et al. [108]. These pharmacogenetic observations may have clinical relevance leading to adverse events (e.g., bradycardia), where those with poor metabolism genotypes have a significantly higher bradycardia event rate relative to those with normal metabolism genotypes [109,110]. Despite CYP2D6 contributing to the majority of metoprolol biotransformation, metoprolol did have a notable stereoselective effect. For example, in a cohort of adults with known *CYP2D6* genotype-dosed metoprolol, those with heterozygous (e.g., CYP2D6*1/CYP2D6*10) and homozygous variant (e.g., CYP2D6*10/CYP2D6*10) genotypes had a ~35% and 154% increase in the AUC of S(−) metoprolol, respectively, and a ~45% and 216% increase in the AUC of R(+) metoprolol, respectively, compared to the reference genotype [108]. As such, the aforementioned results would be expected, as a majority of metoprolol metabolism is derived via o-demethylation, which is preferential to the R(+) enantiomer [73,74]. Propranolol systemic exposure was ~49% and 137% higher in those with a heterozygous (e.g., CYP2D6*1/CYP2D6*10) and homozygous variant (e.g., CYP2D6*10/CYP2D6*10), respectively, compared to the reference genotype [111]. There is an equivocal amount of data to suggest that the CYP2D6 genotype does influence 4-hydroxypropranolol (4-OH propranolol), a potent metabolite, formation [112,113,114,115,116]; however, the parent drug systemic exposure was not significantly different between CYP2D6 genotypes [113,114,116]. Clinically, these differences in 4-OH propranolol formation did not result in an enhanced beta blockade or in adverse events [112,113,114,116]. CYP1A2 maintains an important role in carvedilol and propranolol disposition. However, there is a paucity of data regarding the *CYP1A2* genotype and carvedilol and propranolol. The evidence regarding genetic variation in phase 2 metabolizing enzymes (e.g., UGTs) is very limited. While it is known that carvedilol is a substrate of UGT1A1, UGT2B4, and UGT2B7 [86,87,117], there is equivocal data to inform prescribers if those with genetic variation associated with intermediate or poor metabolism (e.g., UGT1A1*6, *28, *37) result in different outcomes with regards to the response [118]. However, in a small cohort of adult patients (n = 46) with angina pectoralis dosed carvedilol, there was a nearly eight-fold decrease in glucuronidation in patients with at least four mutant alleles (UGT1A1*6, UGT2B7*3, and CYP2D6*10) compared to those with no mutant alleles [105]. Of note, UGT1A1*28 did not have an influence on glucuronidation in this cohort [119]. The influence of *UGT* and *SULT* polymorphisms in those dosed with propranolol has not been previously assessed but should be addressed with future investigations.

Finally, efflux transporters of relevance for atenolol and carvedilol may have a complementary role in the distribution or clearance of these two agents as noted above. Genetic variation of *ABCB1* (encoding for MDR1 or P-Gp), ABCC2 (encoding for MRP2), and *SLC47A1/2* (encoding for MATE1/2) has been well described previously and has functional consequences for several substrates [120,121,122]. However, to date, there are no associative findings between atenolol and/or carvedilol and the gene variants of these efflux transporters.

## 12. Genetic Determinants of Beta Blocker Response

**Figure 3:**
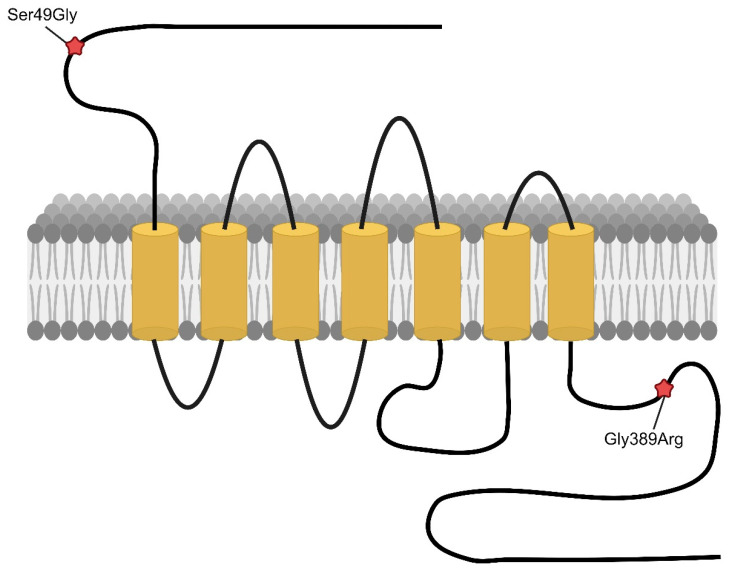
**Adrenergic Beta Receptor Type 1.** Structure and sites of significant polymorphism. Figure created with Biorender.com.

Genetic variation in drug metabolism or response pathways may contribute to interindividual variability observed in drug response. Polymorphisms in the adrenergic signaling system have been associated with drug response [123,124,125], and there are known polymorphisms of ADRB1, some with reported associations with human disease [92]. The two most studied ADRB1 polymorphisms include the nonsynonymous variants: rs1801252, encoding ADRB1-Ser49Gly, and rs1801253, encoding ADRB1-Arg389Gly (Figure 3). The Arg389Gly variant is located within the fourth intracellular loop—this area is highly conserved and important in G-protein coupling, whereas the Ser49Gly variant is located at the extracellular amino terminus of the receptor [92]. Both variants are associated with clinical response to beta blocker therapy, although there are conflicting data on the effect size and direction of effect [124,126,127,128,129,130,131,132,133,134,135].

The functional impact of the rs1801253 variant on ADRB1 function has been assessed. The C>G missense variant leads to a single amino acid substitution, Arg389Gly, which decreases G-protein coupling [136]. The allele frequency of Gly389 varies between white subjects (~27%) and black subjects (~42%) [130]. Initial studies using animal models have shown that the Arg389 receptor signals through its G protein (Gs) more readily than Gly389 receptors [136]. Individuals without this variant, i.e., those with the CC genotype, encoding two copies of the more functional Arg389 protein, would be expected to have a more robust response to beta receptor stimulation using catecholamines and thus blockades as well. Indeed, the rs1801253 CC genotype has been shown to be associated with an increased response to catecholamine stimulation beta blockers in healthy individuals, those with essential hypertension, and heart failure [124,126,127,129,130,131,132,133,134,135,137,138]. There have, however, been negative studies published, particularly in heart failure [118,133,139,140,141,142]. There are also reports of increased response to rate control in atrial fibrillation patients with the CG or GG genotype, rather than the CC genotype [132,133]. The impact of this known genetic variation informed the study published in 2020 by Van Driest, who investigated the response to atenolol or losartan therapy in cases of Marfan syndrome to determine whether variants in ADRB1 (or CYP2C9 for losartan) could identify subgroups of individuals with superior response to either atenolol or losartan [36]. Prior to this, the Pediatric Health Network study compared atenolol with losartan in children and young adults with Marfan syndrome and found no difference in aortic dilation between the two treatment groups [34]. The Van Driest study showed that atenolol-assigned individuals with the ADRB1-rs1801253 CC genotype (encoding Arg/Arg at position 389) had greater improvement in aortic root z-score than those who had CG or GG genotypes (Arg/Gly or Gly/Gly at position 389) [36].

An A→G exchange at codon 49 (Ser49Gly) has an allele frequency of approximately 14% of individuals in various ethnic groups [130]. In vitro, the Gly49 form of the receptor is associated with greater agonist-promoted downregulation [143,144]. The codon 49 and 389 polymorphisms are in linkage disequilibrium [135], i.e., the Gly49Gly389 combination rarely occurs.

***Knowledge of the developmental profile of key pathways involved in beta blocker drug disposition.***

## 13. Developmental Differences in Beta Blocker Disposition

Drugs influenced by physicochemical factors, as noted above (e.g., degree of lipophilicity, extent of protein binding), where known age-dependent changes occur, affecting the volume of distribution, are predictable yet must be taken into consideration when trying to deliver optimal systemic exposure and, as a result, drug response. For example, body water and fat stores are known to change during childhood [10,145], potentially resulting in changes regarding hydrophilic and lipophilic drugs’ volume of distribution, respectively. During the neonatal period, water comprisesapproximately 80% of the total body weight, with a steady decrease in that percentage over the first 4 to 6 months of life, where it approaches normal, healthy adult percentages [146]. Under these neonatal conditions, hydrophilic drugs (e.g., atenolol) would be predicted to have a much larger volume of distribution (e.g., peripheral distribution) and diminished systemic exposure or plasma concentration. Conversely, the total percentage of body fat stores is lower in the neonatal and infancy periods but swiftly rises over the first year of life [145]. Under these neonatal/infancy conditions, lipophilic drugs (e.g., carvedilol, propranolol) would be expected to have a much lower volume of distribution and normal-to-larger systemic exposure. However, data related to the impact of the percentage of adipose tissue (i.e., obesity) and lipophilic drug distribution remains equivocal [147]. Finally, age-related changes in proteins that have the capability to bind drugs can occur. For example, it is known that circulating albumin and α1-acid glycoprotein levels are lowered in neonates relative to adults [47], and these have led to scenarios where a more unbound drug is present in systemic circulation [148,149,150]. Additionally, the reduced binding affinity of certain protein isoforms, namely fetal albumin—which is present in neonates and young infants, can reduce total protein binding as well. Qualitatively, drugs that are extensively protein-bound (e.g., carvedilol, propranolol) can be more sensitive to the aforementioned developmental changes, resulting in a significant increase in free (unbound) drugs and, subsequently, a clinically meaningful impact [10].

As examined above, OCT and OATP transporters, CYP1A2, CYP2D6, and MDR1, have appeared to be the best candidates for targeted evaluation in a pediatric cohort. The development (ontogeny) of drug-metabolizing enzymes (e.g., CYPs) has been well described and is enzyme-specific [151]. More recently, Prasad et al. have characterized the developmental pattern for several of the aforementioned influx and efflux transporters through a proteomic evaluation of human pediatric and adult liver samples [151]. In short, OCT, OATP1B3, and MDR1(P-gp) demonstrate an age-dependent increase in protein expression from the neonatal age compared to adulthood. MDR1 demonstrated an age-dependent increase amongst each age group (neonate to adult, infants to children, infants to adolescents, and infants to adults). OCT1 demonstrated the largest increase from neonates to adulthood with a five-fold increase in protein expression. Conversely, there were no differences in protein expression demonstrated for MATE1 and MRP2, suggesting a rapid maturation of these protein transporters following the neonatal period.

Based on the aforementioned data related to phase 1 and 2 metabolism, CYP1A2, CYP2D6, UGT1A1, UGT1A9, UGT2B4, and UGT2B7 emerged as the most pressing candidates to evaluate for the developing child. CYP2D6 expression is undetectable in the fetus, but maturation is rapid postnatally with the expected maturation of expression and activity in the first several weeks of life [152,153,154]. Conversely, CYP1A2 maturation is delayed through childhood as confirmed by Cazeneuve et al. in a cohort of 34 human livers (fetus, n = 10; neonates, n = 10; infants, n = 9; adults, n = 5), where caffeine, a probe substrate of CYP1A2, metabolism was very low in fetal, neonatal, and infant liver microsomes [155]. Further investigation by Sonnier and Cresteil demonstrated this delayed ontogeny pattern, where children of 1–9 years of age only had ~50–55% of adult levels [156]. Collectively, age-related variability in metabolism might be more impactful for the CYP1A2 substrates relative to CYP2D6.

UGT1A1 increases immediately after birth and achieves adult expression levels at 3 to 6 months of age [157]. Conversely, UGT1A9 had significantly lower expression in infancy, steadily rising through 2 years of age, and not meeting adult expression levels until late childhood [158]. In the same cohort, UGT2B4 has no differences in expression from infancy to 2 years of age, with all having only a 30–40% expression of UGT2B4 relative to adults [158]. UGT2B7 is detectable in the fetus, albeit only 10–20% of that of adults, with adult levels being achieved at 2 to 3 months of age [159]. All these data may be influential in those patients administered carvedilol or propranolol at a young age.

## 14. Developmental Differences in ADRB1

Limited information is available regarding the ontogeny of the mammalian, specifically human, cardiac βadrenergic receptor. The majority of available information stems from animal models, with the majority of studies focusing on embryologic development; post-natal and ontogeny across the lifetime is less known. Using mouse myocardial tissue, Chen et al. [160] showed that the β-adrenergic receptor appears prior to a detectable heart rate response during gestation, which increases significantly during the third trimester (which parallels the increased adrenergic responsiveness) and then dramatically increases in the post-natal period before declining to the adult level. This group then showed, in combination with measurements of adenylate cyclase activity, that β-adrenergic receptors (and adenylate cyclase activity) are present early in gestation [161]. Similar ontogenic findings have been noted in rat models [162].

While there is limited information regarding human cardiac β-adrenergic receptor ontogeny, there have been studies conducted on human cardiac tissue, looking at β adrenergic receptor expression patterns on cardiac myocytes. Human cardiac myocardial tissue contains β1 and β2 adrenergic receptor subtypes in the ventricular myocardium and atrial tissue [93]. These findings differ from that of other types of mammalian myocardium, where β2 receptors, generally, have not been found in the ventricular myocardium, and only small percentages of β2 receptors have been seen in the atrial tissue of some mammalian species in the laboratory [93].

Prolonged stimulation of the β1 receptors, and, to a lesser extent, the β2 receptor, leads to cardiac remodeling. In a study by Bristow published in 1986, the β1 receptor was shown to predominate in the non-failing human ventricle; however, in the failing ventricle, there was a downregulation of β1 receptors with little or no change in the β2 receptor population [93]. A more recent study utilizing explanted hearts from children and adults with idiopathic dilated cardiomyopathy published in 2014 by Miyamoto et al. [163] showed that pediatric-dilated cardiomyopathy patients (which is the most common form of cardiomyopathy in pediatrics) have a unique pattern of β-adrenergic receptor adaptation compared to their adult counterparts. Both populations demonstrated a decrease in total β-adrenergic receptor expression; however, in adult hearts, this decrease was due entirely to the downregulation of β1 receptors, while children had a downregulation of both β1 and β2 receptors. This plays a role in heart failure (and, subsequently, heart failure management via antagonism of the β adrenergic receptor), as there is reduced responsiveness to β adrenergic receptors, due in part to the downregulation and sequestration of receptors [164]. While chronic β1 receptor activation in heart failure results in deleterious processes, such as pathologic gene expression changes and ventricular remodeling, the β2 receptor seems to be beneficial in mediating a pro-survival, anti-apoptotic pathway [165,166]. This human tissue data coupled with experimental models in mice suggest that differences in β2 receptor adaptation may contribute to different responses to therapeutic beta blockades in children versus adults [163,167].

A study by Bathe-Peters et al. [168] showed that β1 receptors are found at the entire cell surface and in the T-tubules. This is in contrast to β2 receptors, which are exclusively found in the T-tubules. This differential localization may help explain their different physiological functions, despite triggering the same biochemical signal, i.e., an increase in cyclic adenosine monophosphate. Collectively, there exists a knowledge gap regarding the specific ontogeny pattern of β1 and 2 receptors in human cardiac tissue that must be prioritized in future investigations.

## 15. Conclusions

Beta blockers are a class of medication that is widely used in the management of pediatric patients with cardiovascular disease. To date, there are no Clinical Pharmacogenetics Implementation Consortium (CPIC) guidelines to inform atenolol, carvedilol, metoprolol, or propranolol usage in the pediatric population; however, these agents should be considered for prioritization in the future. As with any medication, an understanding of the dose–exposure–response relationship of the administered drug is required to predict a response for a particular dose administered. That understanding is dependent upon knowledge of the unique drug properties (e.g., protein binding affinity coefficient, physicochemical properties, drug preparation) and patient factors (e.g., age, underlying cardiac pathophysiology, variable drug metabolism or transport) that contribute to the drug’s “systemic exposure” for the individual child. Adding to this complexity is the contribution of ontogenic factors to beta adrenergic receptor expression and influence on the drug disposition pathway, genetic variation towards variability in drug disposition and response, and age-related differences in the pharmacokinetics of drugs in children. The majority of data that informs beta blocker usage in children stems from studies performed in adults; however, we have seen that there are key differences in the etiology of cardiovascular disease in adults versus children, beta adrenergic expression in these populations, and responses to this class of medication. As we have demonstrated, there remain several knowledge gaps related to the contributions of ontogeny and genetic variation in beta blocker disposition and response in pediatric patients. Starting at the target site (i.e., the cardiac myocyte), further investigations may focus on the pharmacogenomic effects of polymorphisms in the ADRB1 and their relation to ontogeny and the expression of ADRB1 in human pediatric cardiac tissue.

## Figures and Tables

**Table 1:** Commonly utilized pediatric beta blockers drug distribution pathways.

Beta Blocker (Year of Approval)	Absorption	Distribution: Hepatic/Renal Uptake	Metabolism Phase 1	Metabolism Phase 2	Excretion: Efflux
Atenolol (1975)	OATP1A2 OATP2B1	OCT1 (liver) OCT2 (kidney)	Minor	None	MATE1 MATE2 MDR1
Carvedilol (1995)	Passive diffusion	Passive diffusion	CYP2D6 (major R+ enantiomer) CYP1A2 (major S− enantiomer) CYP3A4 (minor both R+ and S−) CYP2C9 (minor R+ enantiomer)	UGT1A1 UGT2B4 UGT2B7	MDR1 (P-gp) MRP2
Metoprolol (1978)	Passive diffusion	Passive diffusion	CYP2D6 CYP2B6 (minor) CYP2C9 (minor) CYP3A4 (minor)	Unknown	Unknown
Propranolol (1964)	Passive diffusion	Passive diffusion	CYP1A2 (major: side chain oxidation; minor: ring oxidation) CYP2D6 (major: ring oxidation; (minor: side chain oxidation)	UGT1A9 UGT2B4 UGT2B7 UGT1A10 (enterocyte) SULT1A3	Unknown

## Abbreviations

ADRB1—β1 adrenergic receptor, ALDH2—mitochondrial aldehyde dehydrogenase, ATP—adenosine triphosphate, AUC—area under the curve, CaMKII—calmodulin-dependent protein kinase II, cAMP—cyclic adenosine monophosphate, CPIC—Clinical Pharmacogenetics Implementation Consortium, CYP—cytochrome P450, MAOA—monoamine oxidase, MATE—Multidrug and Toxin Extrusion, MDR1—Multidrug Resistance Protein 1, MRP2—multidrug resistance-associated protein 2, OATP—organic anion transporter polypeptides, OCT—organic cation transporter, P-gp—P-glycoprotein, P2RY_12_—P2Y purinoreceptor 12, SLCO—solute carrier organic anion transporter, SVT—supraventricular tachycardia, UGT—5′-diphospho glucuronosyl transferases.
